# Medical Diagnosis—A Manual of Clinical Methods

**Published:** 1888-12

**Authors:** 


### BOOK NOTICES AND REVIEWS.

### Medical Diagnosis—A Manual of Clinical Methods. By J. Graham Brown, M. D. Second edition; pages, 285. New York city: E. B. Treat & Co. 1888.

The numerous volumes which are published from time to time upon diagnosis and kindred clinical topics, tend to prove the great value placed upon this branch of medicine by the medical men of to-day Each era in medicine has its fads; the especial fads of to-day in medicine are pathology and diagnosis. But neither of these is at all the exact science which it is claimed to be. Both are useful and both are extremely fallacious, if used improperly. Dr. Brown, in the introduction to his work, tells us that "without accurate diagnosis there can be no rational treatment." We beg leave to differ with Dr. Brown, or at least to suggest to him that there must be very little "accurate treatment" in his school!

Dr. Brown is an Edinburgh physician, and so it is to be presumed he reflects in his work the views of that school of medicine. The volume is an abbreviated review of the general features of clinical diagnosis. To those physicians who like to know the views of different teachers, this volume will be of interest.

